# OTX2 impedes self–renewal of porcine iPS cells through downregulation of NANOG expression

**DOI:** 10.1038/cddiscovery.2016.90

**Published:** 2016-12-05

**Authors:** Ning Wang, Yaxian Wang, Youlong Xie, Huayan Wang

**Affiliations:** 1 Department of Animal Biotechnology, College of Veterinary Medicine, Northwest A&F University, Yangling, Shaanxi 712100, China

## Abstract

The transcription factor Otx2 acts as a negative switch in the regulation of transition from naive to primed pluripotency in mouse pluripotent stem cells. However, the molecular features and function of porcine OTX2 have not been well elucidated in porcine-induced pluripotent stem cells (piPSCs). By studying high-throughput transcriptome sequencing and interfering endogenous *OTX2* expression, we demonstrate that OTX2 is able to downgrade the self-renewal of piPSCs. *OTX2* is highly expressed in porcine brain, reproductive tissues, and preimplantation embryos, but is undetectable in fibroblasts and most somatic tissues. However, the known piPSC lines reported previously produced different levels of OTX2 depending on the induction procedures and culture conditions. Overexpression of porcine OTX2 can reduce the percentage of alkaline phosphatase-positive colonies and downregulate *NANOG* and *OCT4* expression. In contrast, knockdown of *OTX2* can significantly increase endogenous expressions of *NANOG*, *OCT4*, and *ESRRB*, and stabilize the pluripotent state of piPSCs. On the other hand, NANOG can directly bind to the *OTX2* promoter as shown in ChIP-seq data and repress *OTX2* promoter activity in a dose-dependent manner. These observations indicate that OTX2 and NANOG can form a negative feedback circuitry to regulate the pluripotency of porcine iPS cells.

## Introduction

Pluripotent stem cells have two pluripotency states, naive and primed, depending on the origin of cells and the culture conditions.^1^ In mouse, embryonic stem cells (ESCs) derived from the inner cell mass in the early stage of the blastocyst retain naive pluripotency, whereas epiblast stem cells derived from the epiblast in the late stage of the blastocyst retain primed pluripotency.^1–3^ Clear distinctions between the two pluripotent states have been defined on the basis of cell morphology, cytokine supplementation, signaling pathways, formation of chimera and germline transmission *in vivo*, and transcriptomics. Naive pluripotent stem cells show compact and dome-like colonies, depend on leukemia inhibitory factor (LIF), and are able to generate germline transmitting chimeras.^1,2,4,5^ In contrast, primed pluripotent stem cells form flattened colonies, depend on basic fibroblast growth factor (bFGF), and show very limited capacity to generate chimeric offspring.^2,6,7^ Furthermore, a set of transcription factors (TF), such as Nanog, Oct4, Esrrb, and Otx2, has been shown to regulate the transaction between naive and primed pluripotent states.^1,8^

The TF Otx2, which plays an essential role in the regulation of brain and sense organ development and neuronal differentiation,^9–11^ is a crucial regulator in the transition of murine naive ESCs into primed epiblast stem cells.^12^ Increasing the activity of Otx2 causes a significant enrichment of FGF protein expression and reduces the generation of chimeric embryos. Conversely, absence of Otx2 leads to an increase of LIF/STAT3 signaling activity.^12^ Genome-wide mapping of enhancer activity and protein–DNA interaction profiles show that Otx2, as a cell-state-specific regulator, can interact with Oct4 and combine with primed-dominant enhancers to drive the reorganization of enhancer usages during differentiation.^13^ Therefore, Otx2/Oct4-bound enhancers are important for maintaining cellular identity and leading pluripotent stem cells to exit from naive state pluripotency.^14^ In addition, knockdown of *Otx2* leads the primed state cells to flip to naive ESCs by increasing the expression of Esrrb that is directly regulated by Nanog, and Esrrb can form a binding partner with Oct4 to bind to naive-dominant enhancers.^14,15^ Thus, Otx2 may have a mutually exclusive effect on Nanog, which is well studied as a critical factor on blocking the differentiation of pluripotent stem cells^16,17^ and maintaining the pluripotent state of stem cells.^12^

Porcine-induced pluripotent stem cells (piPSCs) have been reported by several laboratories worldwide.^18–24^ Some piPSC lines showed the primed state with bFGF-dependence and mouse epiblast stem cells-like morphology;^18–21^ other piPSC lines showed the naive-like state with LIF-dependence and mouse ESC-like morphology.^23^ Besides, piPSCs cultured with both LIF and bFGF represented the metastable state.^24^ So far, porcine ESCs and naive state piPSCs are difficult to generate. The underlying problems are improper culture conditions used to generate the piPSCs and unclear cell-state-specific regulatory circuitries. Thus, naive piPSC generation will benefit from an understanding of the genetic and epigenetic mechanisms that control the self-renewal and differentiation of piPSCs.

In our previous studies, mRNA expression profiles showed clear differences in the expression status of TFs in LIF-dependent,^23^ bFGF-dependent,^18^ and LIF/bFGF-dependent piPSCs.^24^ Interestingly, *OTX2* expression in LIF-dependent piPSCs was distinguished from that in bFGF-dependent piPSCs, suggesting that OTX2 might act as a molecular marker to classify the different pluripotent states of pig iPSCs. In this study, we explored whether OTX2 was functionally relevant to the pluripotency of piPSCs. Also, we dissected the relationship between OTX2 and NANOG in such regulation.

## Results

### Porcine *OTX2* expression pattern

**Figure 1:**
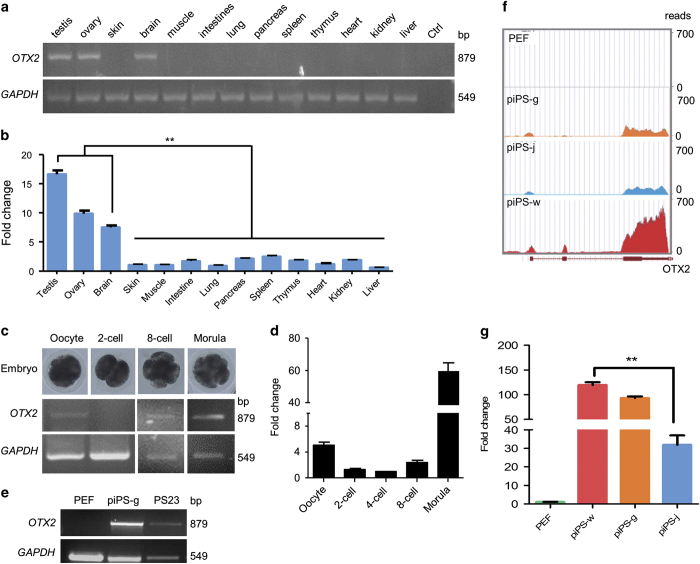
Expression pattern of *OTX2* in porcine tissues and cell lines. *OTX2* expressions in porcine tissues and cell lines were determined by RT-PCR and transcriptome sequencing. (**a** and **b**) *OTX2* expression in porcine tissues. Ctrl, negative control. (**c **and **d**) *OTX2* expression in porcine oocytes and parthenogenetic preimplantation embryos. (**e**) *OTX2* expression in somatic (PEF) and pluripotent (piPS-g and PS23) cells. (**f**) Transcriptome reads of *OTX2* from PEF and porcine iPS cell lines. (**g**) qRT-PCR analysis of *OTX2* expression in PEFs, piPS-w, piPS-g, and piPS-j cells. **P<0.01, *n*=3.

In porcine tissues, including testis, ovary, and brain, *OTX2* was highly expressed (Figure 1a). The qRT-PCR analysis further confirmed that *OTX2* expression in testis, ovary, and brain was 8–17-fold higher than that in other tissues (Figure 1b). Since OTX2 was reported to be relevant to embryo development,^25,26^ we then investigated the *OTX2* expression in porcine embryos. Results of reverse transcription polymerase chain reaction (RT-PCR) and quantitative RT-PCR (qRT-PCR) showed high *OTX2* expression in oocytes, indicating that OTX2 is a maternal factor; *OTX2* expression increased in the preimplantation embryos during the zygotic transition starting from the eight-cell stage (Figures 1c and d). In cell-based experiments, *OTX2* was expressed in porcine LIF/bFGF-dependent iPS cells—piPS-g^24^ and PS23^22^ cells, but not in somatic porcine embryonic fibroblasts (PEF; Figure 1e). This observation allowed us to use these cell lines to explore the function of *OTX2*. RNA-seq data (E-MTAB-2634) showed that *OTX2* was highly expressed in bFGF-dependent iPS cells (piPS-w),^18^ but *OTX2* expression was rather low in LIF-dependent iPS cells (piPS-j).^23^ In LIF/bFGF-dependent iPS cells (piPS-g), *OTX2* expression fell somewhere between piPS-w and piPS-j (Figure 1f). Expression patterns of endogenous *OTX2* in piPSCs were confirmed by qRT-PCR (Figure 1g).

### Characterization of porcine OTX2

**Figure 2:**
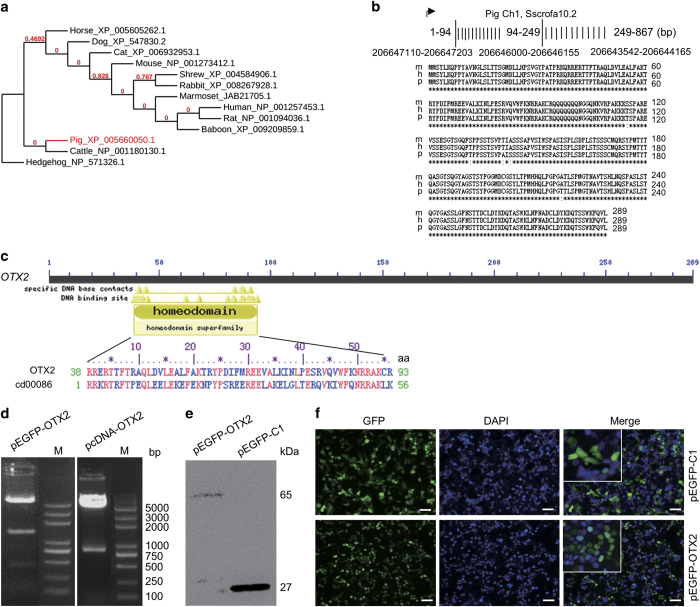
Characterization of porcine OTX2. (**a**) Phylogenetic tree of OTX2 protein calculated by online phylogenetics software. (**b**) Alignment of OTX2 protein sequences among animal species mouse (m), human (h), and pig (p). (**c**) Conserved domain of porcine OTX2 that retains a homeodomain sequence with 56 amino acids. Cd00086 denotes the accession number of the homeodomain sequence in the Conserved Domain Database. (**d**) Restriction enzyme digestions of pEGFP-OTX2 and pcDNA-OTX2. M, D2000 plus DNA ladder. (**e**) Western blotting analysis of GFP protein (27 kDa) and GFP-OTX2 fusion protein (65 kDa) in HEK-293 T cells. (**f**) GFP fluorescent assay. After a 36-h transfection, GFP- and GFP-OTX2-positive cells were observed by fluorescence microscopy. The inset is the enlarged image. Nuclei were stained with DAPI (blue). Scale bar, 50 *μ*m.

Phylogenetic analysis showed that pig OTX2 was evolutionarily related to cattle OTX2, whereas human, baboon, marmoset, rat, and mouse shared the most Otx2 sequence identity (Figure 2a). Porcine *OTX2* CDS was amplified from porcine brain tissue in which OTX2 is an essential factor for brain development,^12^ confirmed by DNA sequencing, and submitted to GenBank (Accession No. KP779653). BLAST research found that the porcine *OTX2* gene was located on the complementary strand of chromosome 1 and contained three exons (Figure 2b). Alignment of the cloned *OTX2* CDS with the pig genome sequence showed 99% identity (Supplementary Figure S1). The 2 kb *OTX2* promoter fragment and an 892 bp fragment of 3′ untranslated region (3′ UTR) were also cloned from porcine brain tissue, and confirmed by DNA sequencing (Supplementary Figure S2). The porcine *OTX2* promoter sequence was submitted to GenBank (Accession No. KR135411).

The protein sequence of OTX2 has 288 amino acids (aa) and exhibits high homology with human and mouse Otx2 (Figure 2b). On the basis of the Conserved Domain Database,^27,28^ OTX2 belongs to the OTX TF super family (Accession No. pfam03529). OTX2 has a homeodomain sequence with 56 amino acids (Accession No. cd00086; Figure 2c), which is predicted to be involved in DNA binding and homodimer or heterodimer formation in a sequence-specific manner.

To investigate the function of OTX2 in eukaryotic cells, we constructed the expression vectors pEGFP-OTX2 and pcDNA-OTX2, which were confirmed by enzyme digestion (Figure 2d). The pEGFP-OTX2 and a control pEGFP-C1 were transfected into HEK-293 T cells for 36 h, and the presence of EGFP-OTX2 fusion protein was confirmed by western blotting (Figure 2e). In a cell-based assay, EGFP-positive and EGFP-OTX2-positive cells were observed with fluorescence microscopy (Figure 2f). EGFP-OTX2 fusion protein was translocated in nuclei, whereas EGFP protein was displayed in whole cells (Figure 2f, inset). Thus, the cloned *OTX2* CDS can be transcribed into functional protein and be used for further research.

### OTX2 influences morphology and expression of pluripotent genes in piPSCs

**Figure 3:**
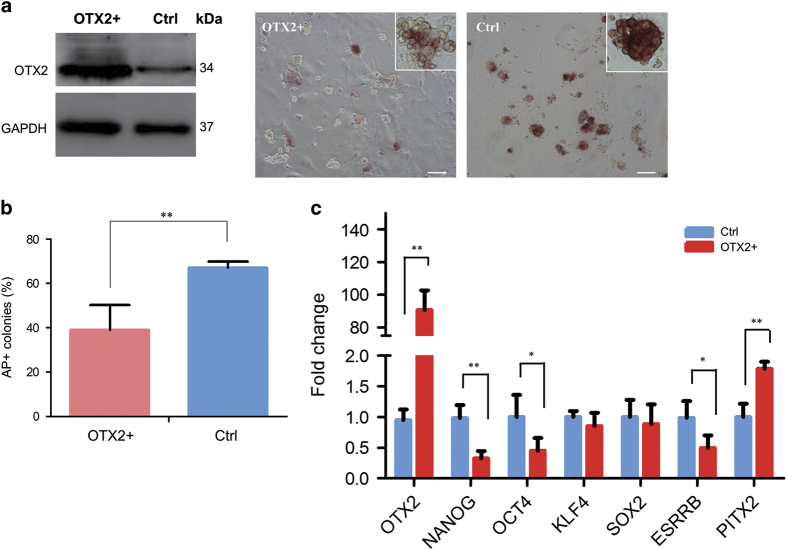
Overexpression of OTX2 in piPSCs. (**a**) Western blotting analysis of OTX2 protein expression and AP staining in OTX2 transfected PS23 cells. Scale bar, 50 *μ*m. (**b**) Quantitative analysis of AP-positive colonies. (**c**) Quantitative RT-PCR analysis of pluripotent gene expressions in PS23 cells. OTX2+, piPS cells were transfected with pcDNA-OTX2; Ctrl, piPS cells without the pcDNA-OTX2 transfection. *P<0.05, **P<0.01, *n*=3.

To investigate the regulatory function of OTX2 in piPSCs, the piPS PS23 cells were transfected by pcDNA-OTX2. Western blotting confirmed that cells transfected with pcDNA-OTX2 expressed high-level OTX2 protein (Figure 3a). Alkaline phosphatase (AP) staining showed that OTX2^+^ piPSC colonies were partially reduced the AP staining compared with the control group, which displayed a more compact morphology with uniform AP staining (Figure 3a). The proportion of AP^+^ colonies in OTX2^+^ piPSCs was significantly lower than that in the control group (Figure 3b), indicating that the pluripotent state of piPSCs was disturbed by ectopic OTX2 overexpression. To further uncover changes in self-renewal, we then examined endogenous pluripotent gene expression in OTX2^+^ piPSCs. The level of *OTX2* in OTX2^+^ piPSCs was significantly increased comparing with the endogenous level of *OTX2* in piPSCs (Figure 3c). However, the expression level of *NANOG* was reduced more than 70% and the expression levels of *OCT4* and *ESRRB* were reduced to 50% in OTX2^+^ piPSCs. Conversely, the expression of *PITX2*, a differentiation-related gene, was significantly increased (Figure 3c). Interference in the expression of other core pluripotent genes, such as *SOX2* and *KLF4*, by OTX2 overexpression was observed, but was not statistically significant (Figure 3c). This observation proved the concept similar to the recently reported results that overexpression of OTX2 significantly downregulated expression of pluripotent genes *NANOG*, *OCT4*, *SOX2*, and so on. and decreased the self-renewal, survival and cell migration in human ES cells.^29^

**Figure 4:**
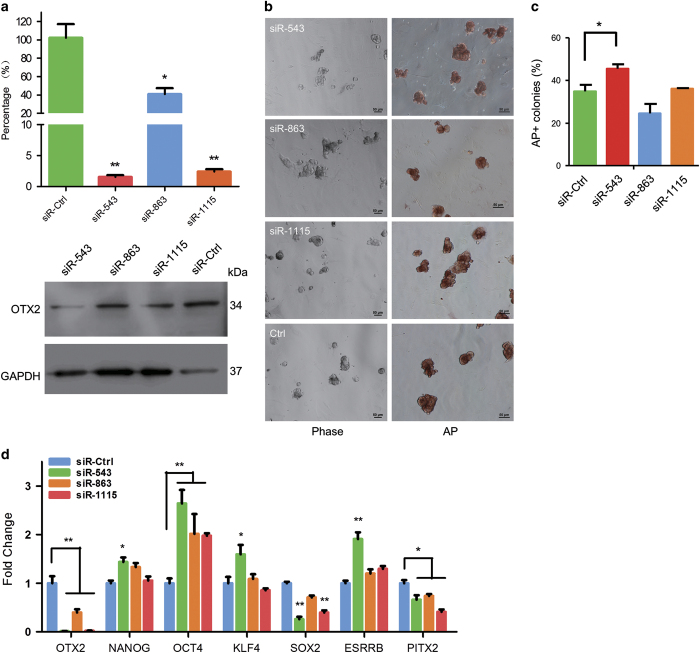
Knockdown of OTX2 expression in piPSCs. (**a**) Quantitative RT-PCR and western blotting analyses of OTX2 expression in PS23 cells that were treated with siRNAs for 36 h. (**b**) AP staining of PS23 cells with siRNAs’ treatment. Scale bar, 50 *μ*m. (**c**) Quantification of AP-positive clones. (**d**) qRT-PCR analysis of pluripotent gene expression in PS23 cells with siRNAs’ treatment. siR-Ctrl, PS23 cells were transfected with control siRNA. *P<0.05, **P<0.01, *n*=3.

The RNA interfering assay showed that expression of OTX2 in both DNA level and protein level was significantly knocked down by all three siRNAs, in which siR-543 and siR-1115 caused 95% reduction in *OTX2* expression (Figure 4a). Besides, the AP staining showed that piPSCs treated with siRNAs exhibited much more compact clones compared with the control group (Figure 4b). Moreover, statistics data showed that the percentage of fine AP-stained colonies in siR-543 treatment was significantly higher than that in a control group (Figure 4c). Additionally, we found that the expressions of endogenous *NANOG*, *OCT4*, and *ESRRB* significantly increased in piPSCs, and *PITX2*, a factor that regulates cell differentiation, was remarkably downregulated in the presence of reduced *OTX2* expression (Figure 4d). Interestingly, *SOX2* expression was reduced significantly when *OTX2* was knocked down, suggesting that further investigation of SOX2 function might reveal its role in regulating the pluripotent state in piPSCs. These observations indicated that *OTX2* knockdown could stabilize the self-renewal and reduce the differentiation potential of piPSCs.

### OTX2 downregulates *NANOG* expression

**Figure 5:**
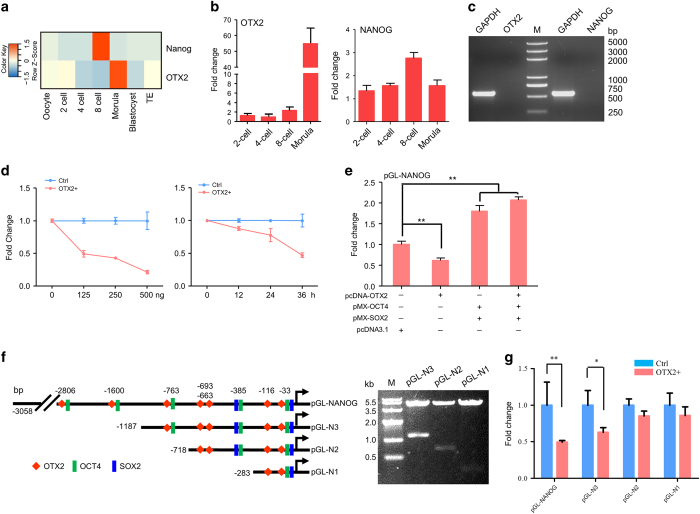
OTX2 downregulates *NANOG* expression. (**a**) Expression profile of *OTX2* and *NANOG* in pig oocyte and parthenogenetic preimplantation embryos. TE, Trophectoderm. (**b**) qRT-PCR analysis of *OTX2* and *NANOG* in two-cell, four-cell, eight-cell, and morula embryos. (**c**) RT-PCR analysis of *OTX2* and *NANOG* in HEK-293 T cells. M, D2000 plus DNA ladder. (**d**) pcDNA-OTX2 and pGL-NANOG were cotransfected into HEK-293 T cells for 36 h. Dose-dependent assay (left) was performed with various concentrations of pcDNA-OTX2. Time-dependent assay (right) was performed with 250 ng of pcDNA-OTX2 for various time points. Ctrl, cells were only transfected with reporter pGL-NANOG. (**e**) Luciferase assay of *NANOG* promoter activation. The expression vectors carrying with *OTX2*, *OCT4*, and *SOX2* were cotransfected with pGL-NANOG into HEK-293 T cells for 36 h, respectively. The pcDNA 3.1 vector was used as a control. (**f**) Truncated constructs of the *NANOG* promoter with predicted DNA binding sites were confirmed by HindIII and *Xho* I double digestion. M, DNA Marker IV. (**g**) HEK-293 T cells were cotransfected with pcDNA-OTX2 and truncated *NANOG* promoter vectors for 36 h. *NANOG* promoter activity was determined by the luciferase assay. Ctrl, cells were only transfected with reporter pGL-NANOG. *P<0.05, ** *P*<0.01, *n*=3.

It has been reported in mouse embryos that loss of Otx2 could severely affect *Nanog* expression.^12^ RNA-seq data generated from different stages of porcine embryos showed that endogenous *NANOG* was highly expressed in eight-cell embryos, whereas *OTX2* was highly expressed at the morula stage (Figure 5a).^30^ Transcriptome analysis, confirmed with qRT-PCR, showed that *OTX2* expression increased significantly in the morula stage, and *NANOG* expression at the eight-cell stage was twofold higher than that at four-cell and morula stages (Figure 5b). In previous experiments (Figure 3c), we noticed that endogenous *NANOG* expression was significantly downregulated in OTX2^+^ piPSCs, suggesting that OTX2 might play a role in negatively regulating *NANOG* expression. To determine the effect of OTX2 on *NANOG* expression, the pcDNA-OTX2 and reporter vector pGL-NANOG were transiently cotransfected into HEK-293 T cells in which both *OTX2* and *NANOG* were not expressed (Figure 5c). Luciferase assays showed that the activity of the *NANOG* promoter could be remarkably downregulated by OTX2 in both time-dependent and dose-dependent manners (Figure 5d). To further investigate activation of the *NANOG* promoter, expression vectors carrying *OTX2*, *OCT4*, and *SOX2* were cotransfected with reporter pGL-NANOG into HEK-293 T cells, respectively. Results showed that addition of OCT4 and SOX2 significantly enhanced *NANOG* promoter activity, similar to a previous study.^31^ However, addition of OTX2 alone significantly reduced *NANOG* promoter activity (Figure 5e). To investigate the synergistic effect of combining OTX2 with OCT4/SOX2, we applied OTX2 with OCT4 and SOX2. *NANOG* promoter activity increased slightly compared with the treatment lacking addition of OTX2, but the increase was not statistically significant (Figure 5e). This result suggests that OTX2 and OCT4/SOX2 do not have a synergistic effect on the activity of porcine *NANOG*; however, the exclusive application of OTX2 does impede *NANOG* promoter activation.

To further investigate OTX2 regulation of *NANOG* expression, we constructed a series of reporter constructs of porcine *NANOG*, which retained the truncated promoter sequence reported previously.^31^ Within the promoter sequence, seven putative OTX2 binding sites and multiple putative OCT4 and SOX2 binding sites were found on the basis of the online JASPAR software (Figure 5f, left). Double enzyme digestions confirmed the accuracy of these recombinant vectors (Figure 5f, right). *NANOG* reporter pGL-NANOG and three truncated constructs pGL-N1, pGL-N2, and pGL-N3 were transiently cotransfected with pcDNA-OTX2 into HEK-293 T cells, respectively. The promoter activity was significantly repressed in cells transfected with pGL-N3 and pGL-NANOG, but not in cells with pGL-N1 and pGL-N2 (Figure 5g). These results indicated that OTX2 could block activation of the *NANOG* promoter and its binding sites were probably located in the distal region of the *NANOG* promoter. In the future study, the gel shift assay and ChIP-seq experiment might reveal whether OTX2 binds directly or indirectly to the *NANOG* promoter.

### NANOG directly regulates *OTX2* expression

**Figure 6:**
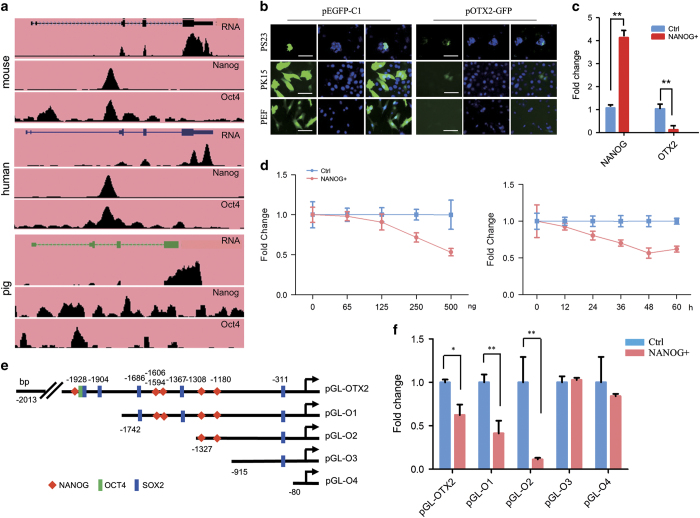
NANOG regulates *OTX2* expression. (**a**) ChIP-seq datasets from mouse, human, and pig show the binding profiles of NANOG and OCT4 associated with the *OTX2* promoter loci. (**b**) GFP fluorescence assay. After 36 h transfection, OTX2-GFP-positive cells were present in PS23 and PK15 cells, but absent in PEF cells. Scale bar, 50 *μ*m. (**c**) qRT-PCR analysis of *NANOG* and *OTX2* expressions in PS23 cells cotransfected with pcDNA-NANOG. (**d**) pcDNA-NANOG and pGL-OTX2 were cotransfected into HEK-293 T cells for 36 h. Dose-dependent assay (left) was performed with various concentrations of pcDNA-NANOG. Time-dependent assay (right) was performed with 250 ng of pcDNA-NANOG for various time points. (**e**) Diagram of reporter constructs with truncated *OTX2* promoter. The predicted binding sites of NANOG, OCT4, and SOX2 are denoted. (**f**) Luciferase assay of OTX2 promoter activity in HEK-293 T cells that were cotransfected with pcDNA-NANOG and a truncated OTX2 vector. Ctrl, cells were transfected only with reporter pGL-OTX2. *P<0.05, **P<0.01, *n*=3.

ChIP-seq datasets from mouse, human, and pig showed that Nanog could directly bind to the mouse and human *Otx2* promoter region. However, pig ChIP-seq profiles exhibited numerous noisy due to the quality of the anti-NANOG antibody that lacks pig specificity (Figure 6a).^32^ To investigate whether NANOG could regulate porcine *OTX2* expression, we cloned the 2 kb porcine *OTX2* promoter and constructed *OTX2* reporter vectors pOTX2-GFP and pGL-OTX2. The pOTX2-GFP and pEGFP-C1 were cotransfected into PEFs, a porcine epithelial cell line (PK15), and a porcine iPS cell line (PS23). Results showed that the *OTX2* promoter was activated in PK15 and PS23 cells, but not in PEF cells, indicating that the cloned *OTX2* promoter retains cell-type specificity (Figure 6b). NANOG overexpression in piPSCs significantly reduced *OTX2* expression (Figure 6c). The dose- and time-dependent assays showed that the activity of the porcine *OTX2* promoter could be repressed by NANOG in a dose-dependent manner (Figure 6d). To further monitor the NANOG binding region, the truncated *OTX2* reporter constructs were made and used for luciferase assays (Figure 6e and Supplementary Figure S3). Within the 2 kb promoter region, there are multiple predicted binding sites of NANOG, OCT4, and SOX2. After removing NANOG binding sites in distal region of *OTX2* promoter, *OTX2* activity was not repressed by NANOG (Figure 6f). These observations indicate that NANOG can negatively regulate activity of porcine *OTX2* promoter. Thus, the binding of NANOG to the *OTX2* promoter could form negative feedback regulatory circuitry to regulate the self-renewal of porcine iPS cells.

## Discussion

As a homeodomain-containing TF in mammals, OTX2 regulation is essential for the normal development of brain and the genesis of photoreceptors.^9–11,33^ Also, Otx2 is described as the marker of the anterior central nervous system.^34,35^ The known Otx2 in several mammals is highly conserved and shares a conserved homeodomain region. High-level expression of *Otx2* was detected in mouse/human brain.^11,36,37^ However, in pig, *OTX2* is highly expressed not only in brain but also in reproductive tissues, as observed in this study. We found that porcine *OTX2* transcripts initially existed in maternal components in oocytes, but were absent at two-cell and four-cell stages; the *OTX2* level increased significantly after the eight-cell stage. In both mouse and human,^25,26^ *Otx2* expression was upregulated along with embryonic development, indicating that OTX2 is an intrinsic TF activated in the stage of zygotic genome activation and determines cell fate in early embryonic development.^33^ We also found that in porcine iPS cell lines, *OTX2* expression was fourfold higher in primed piPSCs than in naive-like piPSCs (Figure 1g). Furthermore, the *OTX2* expression level in metastable state cells was between the levels in naive-like and primed cells. These results indicate that OTX2 can be used as a marker to evaluate the pluripotent state of piPSCs. However, more precise determinations of cell type and culture condition must be applied to verify this hypothesis in future work.

We found that OTX2 and OCT4/SOX2 did not have the synergistic effect on promoting NANOG expression. Alternatively, overexpression of OTX2 alone could repress the expression of pluripotent genes in piPSCs. OTX2 has been shown to bind to the human tenascin-C promoter and transrepress tenascin activity.^38,39^ Investigations of regulatory circuitries of mouse Otx2 and other pluripotent factors have shown that Otx2 can interact with Oct4 and the two proteins cobind to primed-dominant enhancers to activate genes that promote exit from naive pluripotency.^14,40^ As a cell-state-specific regulator, Otx2 dynamically binds to enhancers, H3K4me1 and H3K27ac, that have low histone signals, and opens the previous inaccessible chromatin sites in which Oct4 and other TFs can cobind to these sites and promote enhancer–promoter interactions to elevate the expression of the set of genes that are related to lineage differentiation in primed cells.^13^ Thus, OTX2 is a negative regulator to influence iPS cell pluripotency. We assume that switching off porcine *OTX2* expression during cellular reprogramming may help to gain the naive state piPSCs.

Of note, an abundance or lack of OTX2 in porcine iPS cells repressed and activated the *NANOG* promoter, respectively, in a time- and dose-dependent manner (Figure 5). Studies of single-cell expression profiling under different chemical and genetic perturbations showed that the expression of polycomb target genes, including *Otx2*, existed in a repressed yet poised state with a unique chromatin signature in iPSCs and negatively correlated with *Nanog* expression, representing regulators governing initial steps in lineage commitment.^41^ Similar observations were reported in mouse, in which constitutive and ubiquitous expression of Otx2 leads to a substantial reduction in *Nanog* expression; conversely, reduction of Otx2 causes strong activation of *Nanog.*^12^ In mouse *Otx2*^−/−^ ESCs, cell colonies exhibited a sphere-like morphology, uniform AP staining, ubiquitous distribution of Nanog and Klf4, and a higher abundance Rex1.^12^ In piPSCs, We found that knockdown of *OTX2* with RNA-mediated interference also improved cell morphology and increased the expression of *NANOG*, *OCT4*, and *ESRRB*; however, *KLF4* expression was unchanged and was independent of *OTX2* activation, suggesting that other KLF family members may functionally overlap with KLF4, as reported previously,^24^ to regulate porcine cell reprogramming.

The ChIP-seq result in the pig sample displayed severe noise due to the low quality of the anti-NANOG antibody.^32^ ChIP-seq data from mouse and human showed that NANOG could directly bind to the *Otx2* promoter region, which were also associated with Oct4 binding. A dose-dependent luciferase assay confirmed that NANOG repressed *OTX2* activation (Figure 6). Our findings suggest that OTX2 and NANOG may form negative feedback regulatory circuitry to maintain pluripotent states in piPSCs.

We found that, unlike *OCT4* and *NANOG* expression, *SOX2* expression was positively correlated with *OTX2* expression. Overexpression of *OTX2* slightly elevated *SOX2* expression, however, knockdown of *OTX2* expression significantly reduced SOX2 expression (Figure 4), suggesting that the SOX2 protein, which also functions to specify neural lineage, is a determinant of cellular reprogramming potential and is required for epiblast maintenance.^42,43^ A previous study of Otx2 and Sox2 coregulation of *Rax* gene expression in fog showed that Otx2 overexpression was accompanied by increased expression of Sox2, in which the Otx2 protein directly bound to multiple cis-regulatory elements (N-2, AGATTA; N-3, GGATTA) that spatiotemporally control *Sox2* expression.^44,45^ Thus, the regulatory function of the SOX2 protein in piPSCs may be different from that in mouse pluripotent stem cells. It would be interesting to investigate the transactivation role of SOX2 during the transition of pluripotent states in piPSCs.

In summary, we have identified OTX2 as an important cell-state-specific regulator of the fate and pluripotency of piPSCs, through inhibition of pluripotent gene expressions. We also showed that OTX2 and NANOG exhibited negative feedback circuitry to balance the pluripotency of piPSCs. Further studies will determine the functional relationship between OTX2 and other pluripotent factors and dissect the molecular mechanisms that maintain pluripotency in piPSCs.

## Materials and Methods

### Molecular cloning of *OTX2* and vector construction

Total RNAs were extracted from pig brain tissue by TRIzol Reagent (#15596-026, Invitrogen, Carlsbad, CA, USA) on the basis of the manufacturer’s procedure. Pig *OTX2* coding DNA sequence (CDS) and 3′ UTR sequence were amplified by RT-PCR. The PCR fragments were cloned into the pMD18-T vector (RR420A, Takara, Dalian, China) and confirmed by DNA sequencing. The porcine *OTX2* CDS was submitted to GenBank (Accession No. KP779653). To construct an *OTX2* overexpression vector, the *OTX2* CDS was subcloned into BamHI/SalI sites of pEGFP-C1 (#6084-1, Clontech, Palo Alto, CA, USA) and BamHI/EcoRI sites of pcDNA3.1/V5-His (V385-20, Invitrogen) to generate the recombinant expression vector pEGFP-OTX2 that produces the GFP-OTX2 fusion protein and the recombinant expression vector pcDNA-OTX2 that produces OTX2 protein. To construct the pcDNA-NANOG plasmid, porcine *NANOG* CDS was subcloned into HindIII/XbaI sites of pcDNA3.1/V5-His. *OCT4* and *SOX2* CDSs were subcloned into pMXs vector to generate pMX-OCT4 and pMX-SOX2.

To construct the *OTX2* reporter vector, a 2 kb *OTX2* promoter fragment was amplified by PCR and cloned into pGEM-T Easy vector (A1360, Promega, Madison, WI, USA) and confirmed by DNA sequencing. The porcine *OTX2* promoter sequence was showed in Supplementary Figure S2 and also submitted to GenBank (Accession No. KR135411). The *OTX2* promoter fragment was then subcloned into Xhol/HindIII sites of pEGFP-1 (#6086-1, Clontech) and pGL3-basic (U47295, Promega), respectively. The recombinant vectors were named as pOTX2-GFP and pGL-OTX2. To dissect the core regulatory region of the *OTX2* promoter, we amplified four DNA fragments on the basis of 2 kb *OTX2* promoter sequence and constructed four reporter vectors that retain the truncated *OTX2* promoter. The constructs were confirmed by restricted enzyme digestions (Supplementary Figure S3). The *NANOG* reporter vector pGL-NANOG and a series of deletion constructs, including pGL-N1, pGL-N2, and pGL-N3, were constructed as described previously.^31^

### Cell culture

The porcine iPS cell line PS23 generated in this laboratory was cultured in piPS medium,^22^ which included knock-out DMEM (KO-DMEM, #10829, Invitrogen) supplemented with 20% FBS (#16000-044, Gibco, Grand Island, NY, USA), 0.1 mM nonessential amino acids (NEAA, #11140-050, Invitrogen), 1 mM L-glutamine (#32571-093, Gibco), 10 ng/ml LIF (ESG1106, Millipore, Temecula, CA, USA), 10 ng/ml bFGF (GF003, Millipore), 0.1 mM *β*-mercaptoethanol, 50 units/ml penicillin/streptomycin, at 37 °C, in a 5% CO_2_ humidified atmosphere. PS23 cells were maintained on mitotically inactive mouse embryonic fibroblasts feeder derived from ICR mice, and passaged using 1 mg/ml collagenase type IV (#17104-019, Gibco) and 0.05% trypsin (#27250-018, Gibco) every 2 to 3 days. The mouse embryonic carcinoma cell line P19 was grown in alpha-MEM (#11900-16, Gibco) with 10% FBS (#16000-044, Gibco). HEK-293 T cells were cultured in DMEM with 10% FBS at 37 °C, in a 5% CO_2_ humidified atmosphere. Media were changed every 2 to 3 days.

### Cell transfection

To evaluate *OTX2* expression, cells were seeded in culture dishes 24 h before transfection. After reaching 80% confluence, 4.0 *μ*g pEGFP-OTX2 was transiently transfected into HEK-293 T cells plated on six-well plate using TurboFect transfection reagents (R0532, Thermo, Fair Lawn, NJ, USA) according to the manufacturer’s instruction. After 36 h, GFP-positive cells were examined and collected for western blotting. To determine the tissue specificity of the *OTX2* promoter, the vector pOTX2-GFP was transfected into PEF, PK15, and PS23 cells, respectively, with Lipofectamine 2000 Regent (#11668-019, Invitrogen) for 36 h.

To overexpress *OTX2* in porcine pluripotent stem cells, PS23 cells plated on a six-well plate were transiently transfected with 3.5 *μ*g pcDNA-OTX2 and pEGFP-OTX2, respectively, using Lipofectamine 2000 Regent for 36 h. The transfection efficiency of pEGFP-OTX2 was over 35%. To knockdown *OTX2* expression in piPSCs, three small interfering RNAs (siRNAs; siR-543, siR-863, and siR-1115) and a negative control (siR-Ctrl) were synthesized (GenePharma, Shanghai, China), and the sequence of siRNAs are listed in Table 1. The 200 nM siRNA siR-543, siR-863, siR-1115, and siR-Ctrl were transiently transfected into PS23 cells, respectively, with Lipofectamine 2000 Regent for 36 h. The transfection efficiency of siRNA determined with a fluorescent-labeled control siRNA (GenePharma) was more than 60%. To increase siRNA interference efficiency, a second transfection of siRNA was conducted at 12 h after the first transfection, and samples were analyzed at 24 h after the second transfection.

To investigate NANOG interaction with the *OTX2* promoter, pGL-OTX2, pcDNA-NANOG, and an internal control pRT-TK (0.01 *μ*g) were transiently cotransfected into HEK-293 T cells in a 48-well plate using TurboFect transfection reagents (R0532, Thermo). To determine the OTX2 regulatory effect on the *NANOG* promoter, deletion constructs, including pGL-NANOG, pGL-N1, pGL-N2, and pGL-N3, were transiently cotransfected with pcDNA-OTX2 and pRT-TK into HEK-293 T cells. To explore interactions of OTX2, SOX2, and OCT4 with the *NANOG* promoter, vectors pcDNA-OTX2, pMX-OCT4, and pMX-SOX2 with pGL-NANOG were transiently cotransfected into HEK-293 T cells for 36 h.

### Luciferase assay

After 36 h transfection, cells were collected and lysed for 10 min at room temperature using passive lysis buffer (E194A, Promega). Luciferase activity was detected by luciferase assay reagents (E1960, Promega) and a BHP9504 microplate luminometer (D04407H, Hamamatsu, Beijing, China). Each treatment was measured in triplicate, and the average values of the ratio of firefly luciferase units to renilla luciferase units were used for data analysis. Statistical significance was accepted at *P*<0.05 and determined using the two-tailed *t*-test with equal variance.

### RT-PCR

Total RNA from porcine tissues, pig fibroblasts, piPSCs, and embryos was extracted using TRIzol Reagent (#15596-026, Invitrogen) according to the manufacturer’s protocol. RNA samples were examined by measuring OD260/280 ratio of the optical density. RNAs with an optical density ratio of 2.0 were used for reverse transcription. One microgram of RNA was reverse-transcribed using RevertAid Reverse Transcriptase (EP0732, Thermo). The PCR was performed for 35 cycles at 94 °C 30 s, 56 °C 30 s, and 72 °C 45 s. PCR products were analyzed on 1.0% agarose gel. *GAPDH* was used as an internal control. To obtain the *OTX2* 3′ UTR sequence, 1 *μ*g of RNA was reverse-transcribed using RevertAid Reverse Transcriptase with a 3′ UTR adapter primer. The first PCR reaction was performed for 20 cycles at 94 °C 30 s, 60 °C 30 s, and 72 °C 1 min using the forward GSP1 primer. One microliter of PCR product was then utilized for the nested PCR, which was performed for 30 cycles at 94 °C 30 s, 60 °C 30 s, and 72 °C 1 min, with the forward GSP2 primer. qRT-PCR was performed in triplicate using SYBR Green PCR Master Mix (DRR420, Takara), and products were detected with the CFX96 real-time PCR system (Bio-Rad, Hercules, CA, USA). The reaction condition was: 95 °C, 30 s as the first cycle, and 40 cycles of 95 °C 5 s and 60 °C 30 s. Relative expression levels of genes were normalized to that of *GAPDH* and calculated using 2^–ΔΔCt^. To perform genomic DNA PCR, 1 *μ*g of genomic DNA was extracted from porcine brain tissue using a TIANamp Genomic DNA Kit (DP304-02, Tiangen Biotech, Beijing, China) as the template. PCR reactions were performed for 35 cycles at 94 °C 30 s, 60 °C 30 s, and 72 °C 1 min. PCR products were separated by 1.5% agarose gel electrophoresis. Primers used in this study are listed in Supplementary Table S1.

### Western blotting

To determine the EGFP-OTX2 fusion protein, HEK-293 T cells were transfected with pEGFP-OTX2 and the control vector pEGFP-C1, respectively, using TurboFect transfection reagents (R0532, Thermo) for 36 h. To determine the overexpressed or endogenous OTX2 protein, PS23 cells were transfected with pcDNA-OTX2 and siRNAs, respectively, for 36 h. Cells were then collected by centrifugation. The 1×10^6^ cell pellet was resuspended in SDS-PAGE loading buffer (50 mM Tris-HCl pH 6.8, 2% SDS, 10% glycerin, 2% *β*-mercaptoethanol, and 0.05% bromophenol blue), and heated at 100 °C for 5 min. A 20 *μ*l protein sample was subjected to 12% SDS-PAGE. After electrophoresis, proteins were transferred to a PVDF membrane (LC2002, Invitrogen) by semidry electrophoretic transfer (Bio-Rad) for 45 min at 15 V. The membrane was blocked with blocking buffer (20 mM Tris/HCl pH 7.6, 137 mM NaCl, 0.1% Tween 20, and 8% dried skim milk) at 4 °C overnight, and then incubated with the primary anti-GFP antibody (1 : 5000, KM8009, Sungene Biotech, Tianjin, China), anti-GAPDH antibody (1:3000, KM9002, Sungene Biotech) and anti-OTX2 antibody (1 : 500, bs-11958 R, Sungene Bioss, Beijing, China), respectively, at 37 °C for 2 h. After washing three times with TBS-T buffer (20 mM Tris/HCl pH 7.6, 137 mM NaCl, 0.1% Tween 20), the membrane was incubated with a HRP-conjugated secondary antibody (1 : 3000, A0258, Beyotime, Shanghai, China) at 37 °C for 1 h. After washing in TBS-T for 10 min three times at room temperature, the membrane was incubated in enhanced chemiluminescent substrate (#32106, Pierce, Rockford, IL, USA) for 1 min and detected with a Chemiluminescent ImagingSystem (ZY058176, Tanon-4200, Shanghai, China).

### Alkaline phosphatase staining

To perform AP staining, PS23 cells were washed twice using ice-cold phosphate buffered saline (PBS, pH 7.4), fixed with 4% paraformaldehyde in PBS for 10 min at room temperature, and washed three times using ice-cold PBS. Cells were then incubated at room temperature in 0.1 M Tris buffer, pH 7.4, with 1.0 mg/ml Fast Red TR, 0.4 mg/ml Naphthol AS-MX Phosphate (#1596-56-1, Sigma, St Louis, MO, USA). After 10 min incubation, AP-positive iPS colonies were identified by their red color.

### Data sources and bioinformatics analysis

Protein sequences used in phylogenetic analysis were obtained from the GenBank database. GenBank database for OTX protein is under the following accession numbers: pig, XP_005660050.1; mouse, NP_001273412.1; human, NP_001257453.1; rat, NP_001094036.1; dog, XP_547830.2; cat, XP_006932953.1; horse, XP_005605262.1; cattle, NP_001180130.1; rabbit, XP_008267928.1; shrew, XP_004584906.1; baboon, XP_009209859.1; marmoset, JAB21705.1; and hedgehog, NP_571326.1. ChIP-seq data were from the ChIP-seq dataset (http://www.cepbrowser.org/). ChIP-seq data were obtained from the ChIP-seq dataset.^32^ Pig embryo RNA-seq data were obtained from the Lab Archive (http://www.ncbi.nlm.nih.gov/sra) under accession number SRA076823.^30^ RNA-seq data for piPS-w cells,^18^ piPS-j cells,^23^ and piPS-g cells^24^ were obtained from the EMBL-EBI database (http://www.ebi.ac.uk/) under accession number E-MTAB-2634. Phylogenetic analysis of OTX2 was performed using online software (http://phylogeny.lirmm.fr/phylo_cgi/index.cgi).^46,47^ Genome analysis was performed with the UCSC Genome Browser website (http://genome.ucsc.edu)^48^ and the Ensembl Genome Browser (http://www.ensembl.org/index.html). Multiple sequence alignments were performed by Clustal Omega (http://www.ebi.ac.uk/Tools/msa/clustalo/). Putative sites of TFs were predicted by JASPAR software (http://jaspar.cgb.ki.se/).^49,50^ Conserved domain analysis was performed by the Conserved Domain Database (http://www.ncbi.nlm.nih.gov/Structure/cdd/cdd.shtml).^27,28^

### Statistical analysis

Data are presented as mean±S.D. The S.D. in this study was calculated in three replicates. Statistical significance was assessed using the Student’s *t-*test where indicated in the figure legends.

## Figures and Tables

**Table 1:** siRNAs used in this study

siRNA	Sequence
siR-543	F: 5′- GGGUUCAGGUAUGGUUUAATT-3′
	R: 5′- UUAAACCAUACCUGAACCCTT-3′
siR-863	F: 5′- GGAUAUGCUGGCUCAACUUTT-3′
	R: 5′-AAGUUGAGCCAGCAUAUCCTT-3′
siR-1115	F: 5′- GCUGACUGCUUGGAUUAUATT-3′
	R: 5′-UAUAAUCCAAGCAGUCAGCTT-3′
siR-Ctrl	F: 5′- UUCUCCGAACGUGUCACGUTT-3′
	R: 5′-ACGUGACACGUUCGGAGAATT-3′
