# Supplementary material for: OTX2 impedes self–renewal of porcine iPS cells through downregulation of NANOG expression
Source: Cell Death Discov. 2016 Dec 5;2:16090–. doi: 10.1038/cddiscovery.2016.90 (PMC5136617; doi:10.1038/cddiscovery.2016.90)
Supplement: Supplemental Information [file cddiscovery201690-s1.doc]

**OTX2 Impedes Self-renewal of Porcine iPS Cells through Downregulation of NANOG Expression**

Running title: Porcine OTX2 downregulates NANOG

Ning Wang^+^, Yaxian Wang^+^, Youlong Xie, Huayan Wang*

Department of Animal Biotechnology, College of Veterinary Medicine, Northwest A&F University, Yangling, Shaanxi 712100, China

^+^ These authors contributed equally to this work.

* Correspondence and requests for materials should be addressed to H.W (email: [hhwang101@163.com](mailto:hhwang101@163.com) or [hhwang101@nwsuaf.edu.cn](mailto:hhwang101@nwsuaf.edu.cn))

**Supplementary Information**

**Table S1. Primers used in this study**

| Primers | DNA Sequence (5’-3’) | Size (bp) | Accession No. |
| --- | --- | --- | --- |
| OTX2 | F: TATAGGATCCTGATGTCTTATCTTAAGCAACCG | 879 | KP779653 |
|  | R: TATAGTCGACTCTACAGATCTTCACAAAACCTGG |  |  |
| q-OTX2 | F:CACTGTTTGCTAAGACCCGATACC | 149 | KP779653 |
|  | R: GACCTCCATTCTGCTGTTGTTGC |  |  |
| OTX2 promoter | F: CTCGAGGTGTCAGGCATTTCTAATTGG | 1997 | KR135411 |
|  | R: AAGCTTGCTAAGGTTGTTTGGAGGC |  |  |
| O1 | F: GAAGATCTGGCTTCTCCCTGCCATCTTT | 1865 | KU508422 |
| O2 | R: CCCAAGCTTCGTAAGTGACTCGGTCCGGC | 1450 | KU508422 |
|  | F: GAAGATCTCTTAACACCATTCCCGCCCT |  |  |
| O3 | R: CCCAAGCTTCGTAAGTGACTCGGTCCGGC | 1038 | KU508422 |
|  | F: GAAGATCTGTGGGAACCAGCTCAAGGTT |  |  |
| O4 | R: CCCAAGCTTCGTAAGTGACTCGGTCCGGC | 203 | KU508422 |
|  | F: GAAGATCTACAGCAACTGGTAGATGGGC |  |  |
| NANOG | R: CCCAAGCTTCGTAAGTGACTCGGTCCGGC | 915 | NM_001129971 |
|  | F: GAAGATCTATGAGTGTGGATCCAGCTTGT  R: GGAATTCGATCGTCACATATCTTCAGGCT |  |  |
| q-NANOG | F: TCTCCTCTTCCTTCCTCCAT | 198 | NM_001129971 |
|  | R: CTGCCTCTGAAATCTGTCGT |  |  |
| q-OCT4 | F: GTGTTCAGCCAAACGACCATC | 143 | NM_001113060 |
|  | R: GTCTCTGCCTTGCATATCTCC |  |  |
| q-SOX2 | F: GCCGAGTGGAAACTTTTGTC | 138 | NM_001123197 |
|  | R: CTTCTTCATGAGCGTCTTGGT |  |  |
| q-KLF4 | F: CCATCGGTCATCAGTGTTAGC | 102 | EU669075 |
|  | R: CTTGATCTTGGGGCACATGC |  |  |
| q-ESRRB | F: AGGGAGCTCGTGGTCATCAT | 62 | [XM_001928051](http://www.ncbi.nlm.nih.gov/nucleotide/545842528?report=genbank&log$=nucltop&blast_rank=19&RID=JPSA7D73013).5 |
|  | R: CCCCAGAGAGAGGTTGGAGAA |  |  |
| q-PITX2 | F: ACCCGCTCCCTGGACTCTT | 118 | XM_005666933.1 |
|  | R: CGCCCACATCCTCATTCTTT |  |  |
| GAPDH | F: AAGGTCGGAGTGAACGGATT | 549 | NM_001206359 |
|  | R: AGTCTTCTGGGTGGCAGTGAT |  |  |
| q-GAPDH | F:ACCTGCCGCCTGGAGAAACC | 252 | NM_001206359 |
|  | R:GACCATGAGGTCCACCACCCTG |  |  |
| q-H2a | F: GCTCTTCTAGGGCTGGTCTCC | 183 | XM_001927727.3 |
|  | R: ATGCGGGTCTTCTTGTTGTC |  |  |
| OTX2 3’UTR | GSP1 F: GACTGCTTGGATTATAAAGATC  GSP1 R: CCAGTGAGCAGAGTGACG | 1000 | XM_005659993.1 |
|  | GSP2 F: GTCCCAGGAGGCATAGAAG  GSP2 R: GAGGACTCGAGCTCAAGC | 800 |  |
| 3’UTR Adaptor | R: GCTGTCAACGATACGCTACGTAACGGCATGAC  AGTG(T)_18_ | -- | -- |

* Underlines denote the restricted enzyme sites.


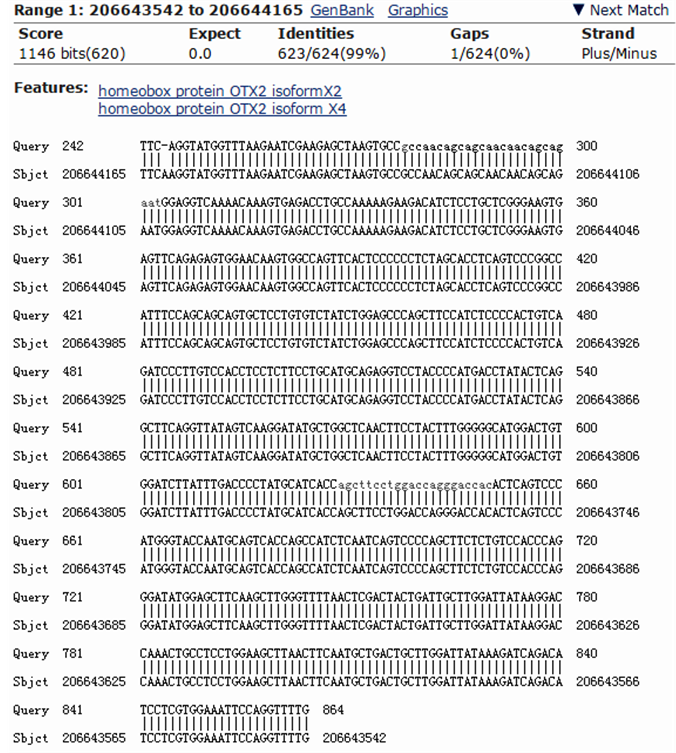


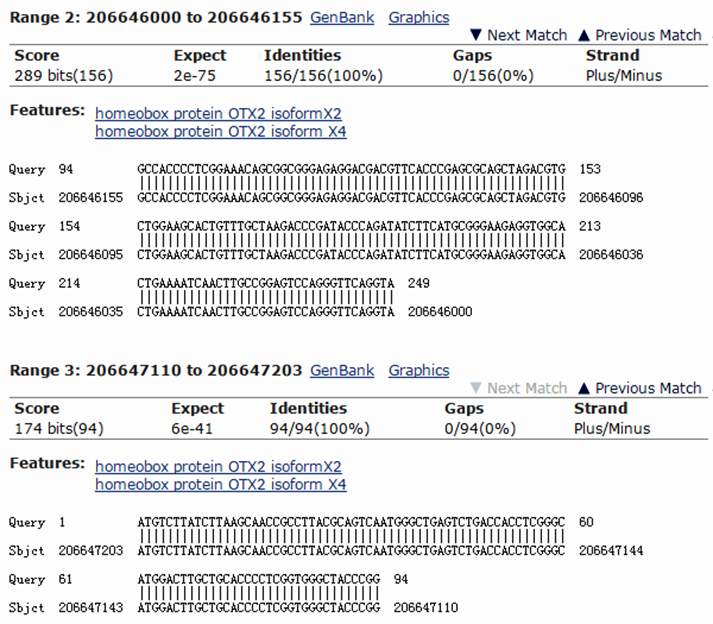


**Figure S1. Alignment of Porcine *OTX2* cDNA Sequence.** Blast analysis of porcine *OTX2* cDNA sequence (Quary) versus pig genome DNA sequence (Subject).

1.
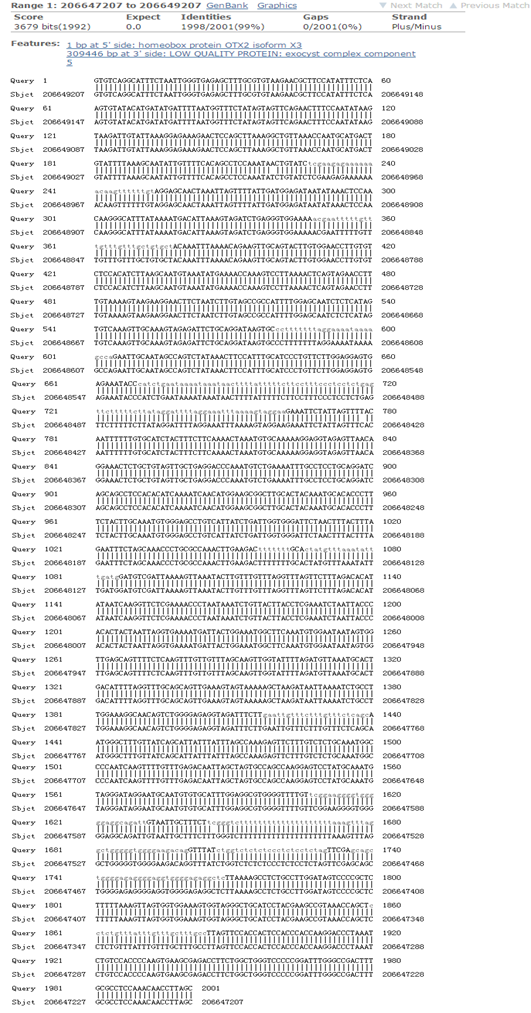

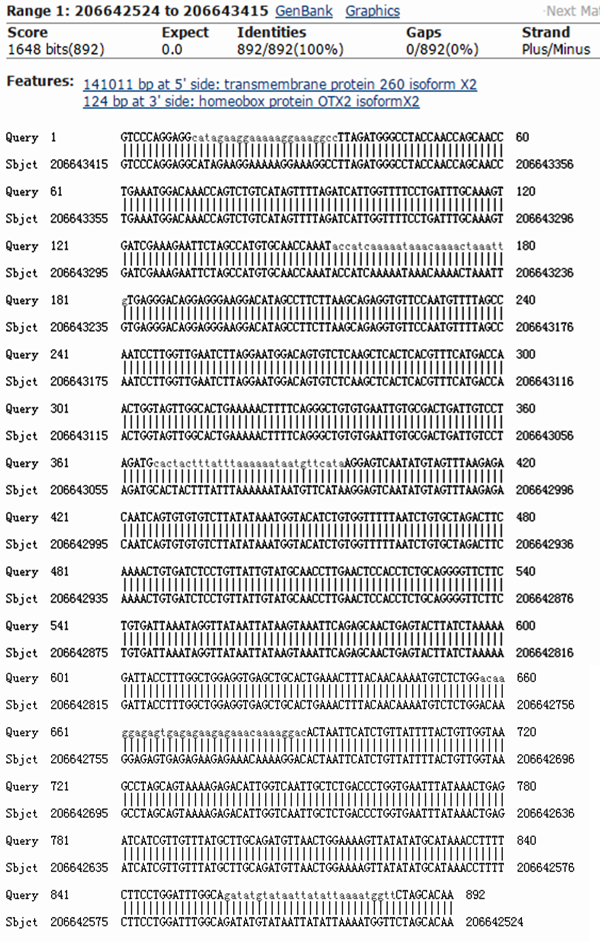
 B.

**Figure S2. Alignment of Porcine *OTX2* Promoter Sequence and 3’ UTR Sequence.** Blast analysis of 2 kb porcine *OTX2* promoter sequence (A) and 892 bp 3’ UTR sequence (B) versus pig genome DNA sequence (Subject).


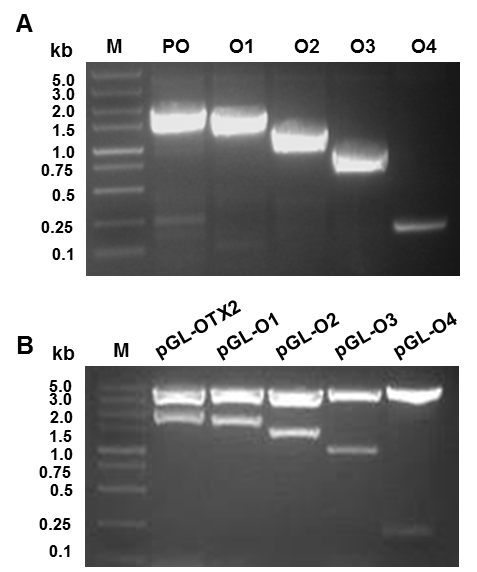


**Figure S3. Construction of porcine OTX2 promoter reporter vectors. A,** PCR products of truncated OTX2 promoter in different sizes. PO, 2. 1 kb; O1, 1.8 kb; O2, 1.4 kb; O3, 1.0 kb; O4, 0.2 kb. **B,** The reporter constructs with truncated OTX2 promoter were digested by Hind III and Bgl II double-digestion. M, DNA Marker DL5000.
